# Co-morbidity of malaria and soil-transmitted helminths in Nigeria: a joint Bayesian modelling approach

**DOI:** 10.1186/s40249-025-01276-x

**Published:** 2025-04-02

**Authors:** Faith Eshofonie, Olatunji Johnson, Ezra Gayawan

**Affiliations:** 1 Department of Statistics, Federal University of Technology, Akure, Nigeria; 2 Department of Mathematics, University of Manchester, Manchester, UK

**Keywords:** Malaria, Soil-transmitted helminths, *Ascaris lumbricoides*, Hookworm, *Trichuris trichiura*, Nigeria, Climate, Coregionalization

## Abstract

**Background:**

Malaria and soil-transmitted helminths (STH) represent significant public health challenges in tropical regions, particularly affecting children and impeding development. This study investigates the co-morbidity of malaria, caused by *Plasmodium* spp., and STH infections, including *Ascaris lumbricoides*(roundworm), *Ancylostoma duodenale* and *Necator americanus* (hookworm), and *Trichuris trichiura*(whipworm), in Nigeria.

**Methods:**

We utilized malaria prevalence data from the Nigeria Malaria Indicators Survey (NMIS) for the years 2010 and 2015 and STH prevalence data from the Expanded Special Project for Elimination of Neglected Tropical Diseases (ESPEN) portal, covering the years 1978–2014. A Bayesian coregionalization model was employed to analyze the prevalence and incidence of malaria and STH, linking these data to climatic factors such as temperature and precipitation. The study’s findings highlight significant co-morbidity between malaria and STH, particularly in the southsouth and southeast regions.

**Results:**

Our analysis reveals notable regional disparities: malaria prevalence is highest in the northwest and north-central regions, while *Ascaris lumbricoides* is widespread in both northern and southern states. *Ancylostoma duodenale and Necator americanus*(Hookworm) are predominantly found in the southwest, and *Trichuris trichiura*, though less prevalent, is significant in specific areas. Substantial co-morbidity between malaria and STH was observed, particularly in the South-South and southeast regions, indicating a compounded health burden. Furthermore, climatic factors significantly influence disease distribution; higher temperatures correlate with increased malaria prevalence, although temperature has a minimal effect on STH prevalence and incidence. In contrast, precipitation is positively associated with both malaria and STH incidence.

**Conclusions:**

These findings enhance our understanding of the spatial distribution and risk factors associated with malaria and STH in Nigeria, providing vital insights for the development of public health policies and targeted intervention strategies.

## Background

Malaria and soil-transmitted helminths (STH) are major parasitic diseases that present significant public health challenges in tropical and subtropical regions [1]. These diseases, along with other neglected tropical diseases (NTDs), disproportionately affect impoverished communities in these regions [2]. Nigeria, the Democratic Republic of Congo, Ethiopia, and Tanzania account for over 50% of Africa’s NTD burden. Nigeria bears the heaviest burden of NTDs in sub-Saharan Africa, with high rates of diseases such as elephantiasis, river blindness, schistosomiasis, and STHs like ascariasis, hookworm, and trichuriasis [3].

STH are caused by nematode worms that spread through soil contaminated with fecal matter. These infections affect over 2 billion people globally, primarily in underprivileged communities lacking access to clean water, sanitation, and hygiene [2]. The major STH parasites include *Ascaris lumbricoides* (roundworm), *Trichuris trichiura* (whipworm), *Necator americanus*, and *Ancylostoma duodenale *(hookworms) [2]. STH infections are widespread in Nigeria, with varying prevalence across all states [4]. Transmission of STH occurs via two primary modes: larvae actively penetrating the skin, as seen in hookworm infections, or ingestion of eggs through contaminated food or water, leading to infections by *Ascaris lumbricoides* and *Trichuris trichiura* [5]. Mild STH infections often exhibit no symptoms, but severe cases can result in anemia, vitamin A deficiency, malnutrition, loss of appetite, and stunted growth [6]. Children infected with STH, as studies show, are particularly at risk of anemia and lower hemoglobin levels compared to their uninfected counterparts [6–8].

Malaria, the most common parasitic disease globally, predominantly affects tropical and subtropical regions. It is mostly caused by *Plasmodium* parasites and transmitted by *Anopheles* mosquitoes [9]. The five *Plasmodium* species infecting humans include *plasmodium falciparum*, *plasmodium malariae*, *plasmodium vivax*, *plasmodium ovale*, and *plasmodium knowlesi*, among these, *plasmodium* falciparum is the most lethal and prevalent in sub-Saharan Africa [10]. Malaria’s impact is especially severe among young children and pregnant women [11]. Symptoms range from mild fever and chills to severe cases leading to organ failure and death [12]. Environmental factors like regional conditions, temperature, and mosquito population influence malaria incidence [13]. Malaria remains a significant health concern, with Nigeria alone accounting for a substantial percentage of the global malaria burden and deaths, particularly among children under five [14]. The World Health Organization reported that in 2022, Nigeria accounted for 38.5% of global malaria deaths in children under five [14].

The rising interest in co-morbidity within epidemiology highlights the need to understand the interplay between malaria and STH, as co-infections can exacerbate health issues like anemia and lead to adverse outcomes such as learning disabilities and higher dropout rates in children [7, 15]. Despite the distinct transmission mechanisms of malaria and STH, their shared environmental, socioeconomic, and biological factors suggest potential epidemiological interactions, contributing to co-infection in a substantial portion of the global population [16]. Previous studies highlight a bidirectional relationship between STH and malaria, impacting susceptibility and clinical outcomes [17–19]. Research by [20] identified co-clustering of malaria and STH using bivariate LISA in sub-Saharan Africa (SSA), highlighting geographical patterns of co-endemicity and showing a significant decrease in prevalence from 2000 to 2018, though high rates remain in West and Central Africa. Studies by [21] and [22] provided insights into STH prevalence in Nigeria, using Bayesian geostatistical models and MaxEnt models, respectively. A decline in malaria was noted by [23] among children under five, while separate studies by [24] and [25] linked co-morbidity of malaria and anemia to urban residency, mother’s education, and household wealth while highlighting the geographical overlap. Despite extensive research on malaria and STH individually, there is a notable gap in studies examining their co-morbidity in Nigeria, where both diseases are endemic. This is particularly concerning given the significant burden of both diseases in Nigeria, where millions are affected. Current control strategies are largely disease-specific, with malaria addressed through insecticide-treated nets (ITNs), indoor residual spraying (IRS) and other vector control measures, while STH is majorly through mass drug administration (MDA). However, these approaches overlook the potential for co-infections among the vulnerable groups. Evidence of shared risk may be useful for coordinated program planning and delivery leading to optimal use of the available meager resources.

Mapping disease prevalence and distribution is a critical tool in epidemiology, as it helps identify geographic clusters, spatial patterns, and high-risk areas, which are essential for targeting interventions and optimizing resource allocation [26]. Traditional univariate mapping methods, which focus on a single disease, have long been used to illustrate the distribution of diseases. However, these methods have limitations, particularly in regions where multiple diseases co-exist. In areas of co-endemicity, where diseases share overlapping risk factors, univariate mapping techniques fail to capture the complex interactions between diseases. This issue is evident in Nigeria, where malaria and STH are often mapped individually. In contrast, multivariate disease mapping helps to simultaneously estimate the risk of multiple diseases in a particular location by leveraging information from related diseases and neighboring areas. As a result, it provides more reliable estimates than traditional univariate disease mapping methods [27]. The Bayesian coregionalization model [28] employed in this study enables the joint modeling of the spatial patterns of malaria and STH by effectively capturing spatial dependencies within each disease and their cross-correlations, allowing for the identification of regions with high risk for malaria, STH, or both diseases.

This research, which examines the co-morbidity of malaria and STH in Nigeria using a Bayesian coregionalization model, investigates the impact of climatic factors on the transmission and prevalence these diseases across the country. The research also explores the spatial distribution of malaria prevalence, STH prevalence, and STH incidence, and identifies geographic hotspots where malaria and STH overlap, indicating areas of high co-morbidity. Understanding these interactions is crucial for devising targeted disease control measures that address the distinct challenges posed by co-infections. The insights gained from this study are anticipated to impact public health interventions significantly, ultimately aiming to alleviate the burden of these diseases and improve health outcomes in Nigeria.

## Methods

### Study area

**Fig. 1:**
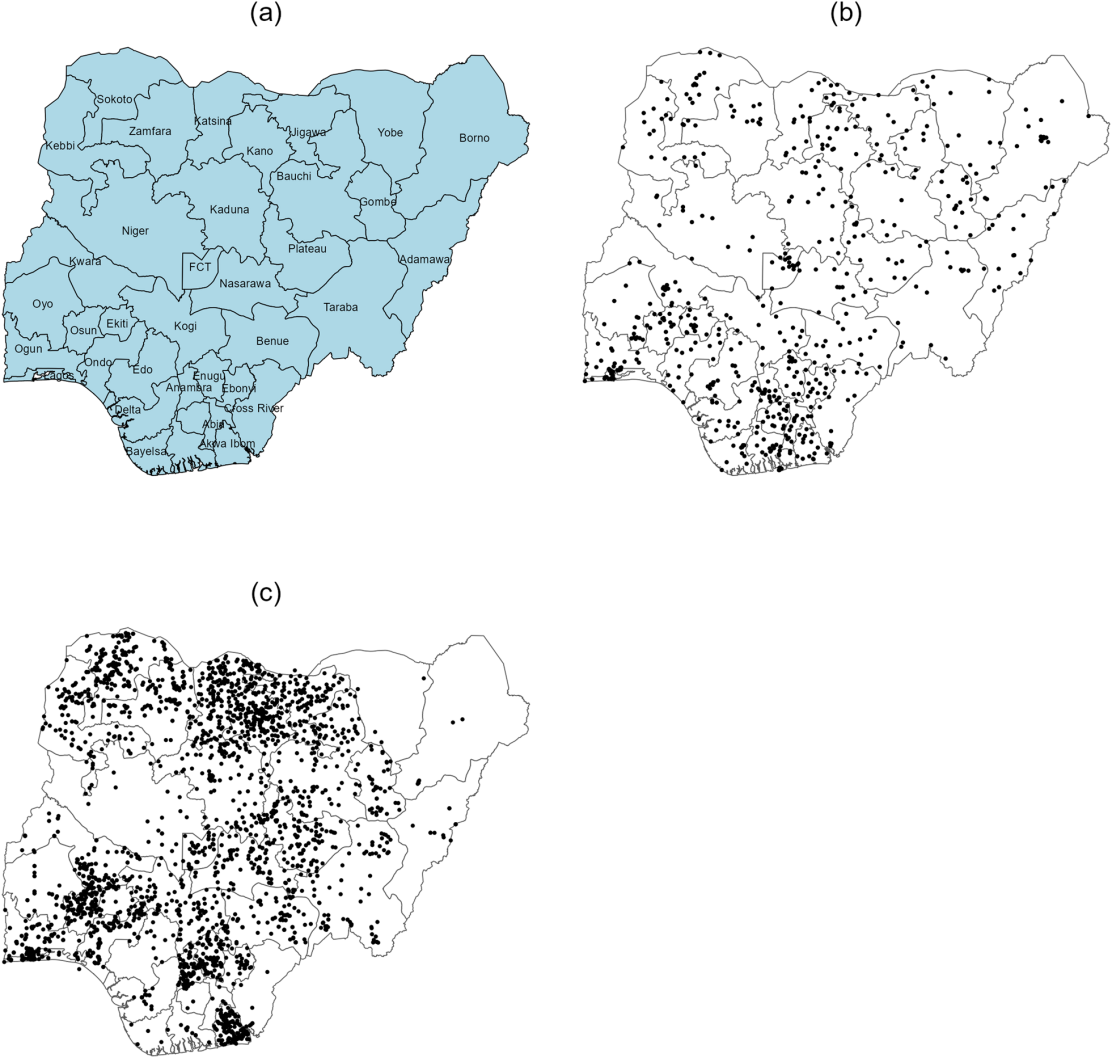
Maps of Nigeria for various data collections. **a** A map of Nigeria showing all 36 states and the FCT. **b** A map of Nigeria showing the locations where malaria prevalence data were collected. **c** A map of Nigeria showing the locations where STH prevalence data were collected

The study focuses on Nigeria, located in West Africa and bordered by Niger to the north, Chad to the northeast, Cameroon to the east, and Benin to the west. Nigeria is administratively divided into 36 states and the Federal Capital Territory, Abuja. Figure 1a provides a map of Nigeria showing all 36 states and the Federal Capital Territory.

### Malaria data

Malaria prevalence data was collected from the Nigeria Malaria Indicators Survey (NMIS) conducted by the National Population Commission (NPopC), the Nigeria Malaria Elimination Programme (NMEP), and the National Bureau of Statistics (NBS). The surveys carried out in 2010 and 2015 [29], provide important information on malaria indicators across all 36 states and the Federal Capital Territory. The NMIS sampling frame was based on the 2006 Population and Housing Census (PHC) of Nigeria, and both surveys employed a two-stage sampling strategy. Further details on the surveys can be found in [30, 31].

Geospatial covariate data were obtained from the Demographic and Health Surveys (DHS) spatial data repository [32]. This dataset includes GPS coordinates for each survey cluster, recorded at the center of the primary sampling unit. To maintain participant confidentiality, the GPS coordinates were adjusted by up to 2 kms in urban areas and up to 10 kms in rural areas. The average parasite rate of *Plasmodium falciparum* (PfPR) in children aged 2–10 years was obtained from estimates provided by the Malaria Atlas Project [33, 34]. Figure 1b shows the locations where malaria prevalence data were collected. The DHS geospatial team standardized files for integrating these data with geospatial covariates derived from both raster and vector sources. Climatic covariates such as temperature and precipitation data were also included in the geospatial dataset to assess their relationship with malaria transmission. Detailed documentation on the DHS geospatial covariate datasets and methodologies is available [35].

### STH Data

STH prevalence is defined as the proportion of children infected with STH such as *Ascaris lumbricoides*, *Trichuris trichiura*, *Ancylostoma duodenale* and *Necator americanus* (hookworms) in a given population at a specific time and location. The data were obtained from the Expanded special project for elimination of neglected tropical diseases (ESPEN) portal [36]. ESPEN Collect is a mobile data collection tool designed for national programs, NTD-related NGOs, and partners in the World Health Organization’s African region. It facilitates data collection for NTDs responsive to preventive chemotherapy, including STH, lymphatic filariasis, onchocerciasis, and schistosomiasis. These surveys typically employ cluster-based sampling methods, with selected communities undergoing parasitological testing, such as stool sample collection for STH. The STH dataset used in this study includes prevalence data for 2905 locations across various states in Nigeria, covering the years 1978–2014 (Fig. 1c).

### STH Incidence data

STH incidence is the rate at which new cases of STH infections occur in a population over a certain period. The data were generated from the STH prevalence dataset. Prevalence values greater than zero were set to 1, indicating the presence of STH infection, while zero prevalence values were set to 0, indicating the absence of infection.

Climatic variables for the STH dataset were obtained from the WorldClim database, which provides monthly climate data for minimum, maximum, and average temperatures, as well as precipitation, solar radiation, wind speed, and water vapor pressure. These data are available at four spatial resolutions ranging from 30 s ( 1 km$^2$) to 10 min ( 340 km$^2$) [37].

**Table 1:** Descriptive statistics for disease prevalence

Disease	Mean	SD
Malaria prevalence	0.295	0.155
*Ascaris lumbricoides* prevalence	0.181	0.188
Hookworm prevalence	0.137	0.316
*Trichuris trichiura* prevalence	0.090	0.124

### Geostatistical model

The Bayesian coregionalization model proposed for multivariate point-referenced data [38] is based on a linear combination of independent spatial processes. The model considers malaria prevalence ($\mu _1(s)$), STH prevalence ($\mu _2(s)$), and STH incidence ($\mu _3(s)$) as the outcomes of interest and linked them to spatial covariates through an additive model, such that:

1 $$\begin{aligned} \mu _1(s)&= \alpha _1 + f(\text {temperature}) + f(\text {precipitation}) + z_1(s) \end{aligned}$$

2 $$\begin{aligned} \mu _2(s)&= \alpha _2 + f(\text {temperature}) + f(\text {precipitation}) + \lambda _1 z_1(s) + z_2(s) \end{aligned}$$

3 $$\begin{aligned} \mu _3(s)&= \alpha _3 + f(\text {temperature}) + f(\text {precipitation}) + \lambda _2 z_1(s) + \lambda _3 z_2(s) + z_3(s) \end{aligned}$$

where $\alpha _k$, $k=1,2,3$ are intercepts specific to each outcome, representing the baseline risk of each disease. $f(\text {temperature})$ is a function representing the non-linear relationship between temperature and the response variable. $f(\text {precipitation})$ is a function representing the non-linear relationship between precipitation and the response variable. The structured spatial random effects $z_k(s)$, $s \in \mathbb {R}^2$ unique to each disease, capture the disease-specific spatial effect at location $s$. These structured spatial effects are shared across the diseases with weights $\lambda _k$ (for $k=1,2,3$) controlling the extent to which the spatial effects of others influence each disease.

The prior distribution for spatial weights was modeled as a Gaussian distribution with a mean of zero and a precision parameter of 10, providing a vague prior. Temperature and precipitation covariates were modeled based on a first-order random walk model. The spatial effects, both shared and non-shared, were modeled using the stochastic partial differential equation (SPDE) method. This SPDE approach, as described by [39] and implemented in the R-INLA software [40], offers a robust framework for spatial data modeling. Typically, Gaussian Markov random fields (GMRFs) are used for discrete spatial domains like lattices and regional adjacency graphs, due to their efficient computation with sparse precision matrices, which are well-suited for large datasets. These spatial relationships are represented by a graph where a precision matrix $\textbf{Q}$ defines dependencies between nodes (spatial locations). This sparse matrix structure ensures that nodes only interact with their neighbors, minimizing computational complexity. A new class of GMRFs with continuous indexing, explicitly mapping Matérn Gaussian fields was introduced by [39], which are widely used in spatial statistical modeling.

For a spatial field *x* at location $\textbf{s} \in \mathbb {R}^2$, GMRFs are generated by solving the stochastic partial differential equation (SPDE):

4 $$\begin{aligned} (\kappa - \Delta )^{\alpha /2} [\tau x(\textbf{s})] = W(\textbf{s}), \end{aligned}$$

where $\Delta$ denotes the Laplacian, $\kappa> 0$ represents the spatial scale, $\alpha> 0$ controls the smoothness, $\tau> 0$ controls the variance, and $W(\textbf{s})$ denotes the Gaussian white noise. The stationary solutions for $x(\textbf{s})$ exhibit Matérn covariance:

5 $$\begin{aligned} \text {Cov}(x(0), x(\textbf{s})) = \sigma ^2 \frac{2^{1-\nu }}{\Gamma (\nu )} (\kappa |\textbf{s}|)^{\nu } K_{\nu }(\kappa |\textbf{s}|) \end{aligned}$$

where $\nu = \alpha - \frac{d}{2}$ influences smoothness, and the marginal variance is given by:

6 $$\begin{aligned} \sigma ^2 = \frac{\Gamma (\nu )}{\Gamma (\alpha ) \left( \frac{4 \pi ^{d/2} \kappa ^{2\nu } \tau ^{2\nu }}{\Gamma (\nu )}\right) } \end{aligned}$$

where $\Gamma (\cdot )$ is the gamma function and $K_{\nu }(\cdot )$ is the modified Bessel function of the second kind.

To approximate the solution of an SPDE, the finite element method was utilized [39]. The domain is divided into “elements,” such as grids or triangulations, known as meshes. Each mesh point $j = 1, \ldots , n$ is associated with a basis function $\psi _j$. The SPDE solution is expressed as a weighted sum of these basis functions and random variables:

7 $$\begin{aligned} x(\textbf{s}) = \sum _{j=1}^{n} \psi _j(\textbf{s}) x_j, \end{aligned}$$

where $\psi _j(\textbf{s})$ are deterministic basis functions, and the joint distribution of the vector $\textbf{x} = (x_1, \ldots , x_n)$ is selected to ensure that the functions $x(\textbf{s})$ approximate the SPDE solutions over the domain.

Hyper priors for the precision parameters are based on penalized complexity (PC) priors introduced by [41]. The expression of the PC-prior is:

$$\begin{aligned} \pi (\tau ) = \lambda ^2 \tau ^{-\frac{3}{2}} \exp \left( -\lambda \tau ^{-\frac{1}{2}}\right) , \quad \tau> 0, \lambda> 0. \end{aligned}$$

The parameter $\lambda$ controls the extent of the penalty applied for deviating from the base model, with larger values resulting in a greater penalty.

**Fig. 2:**
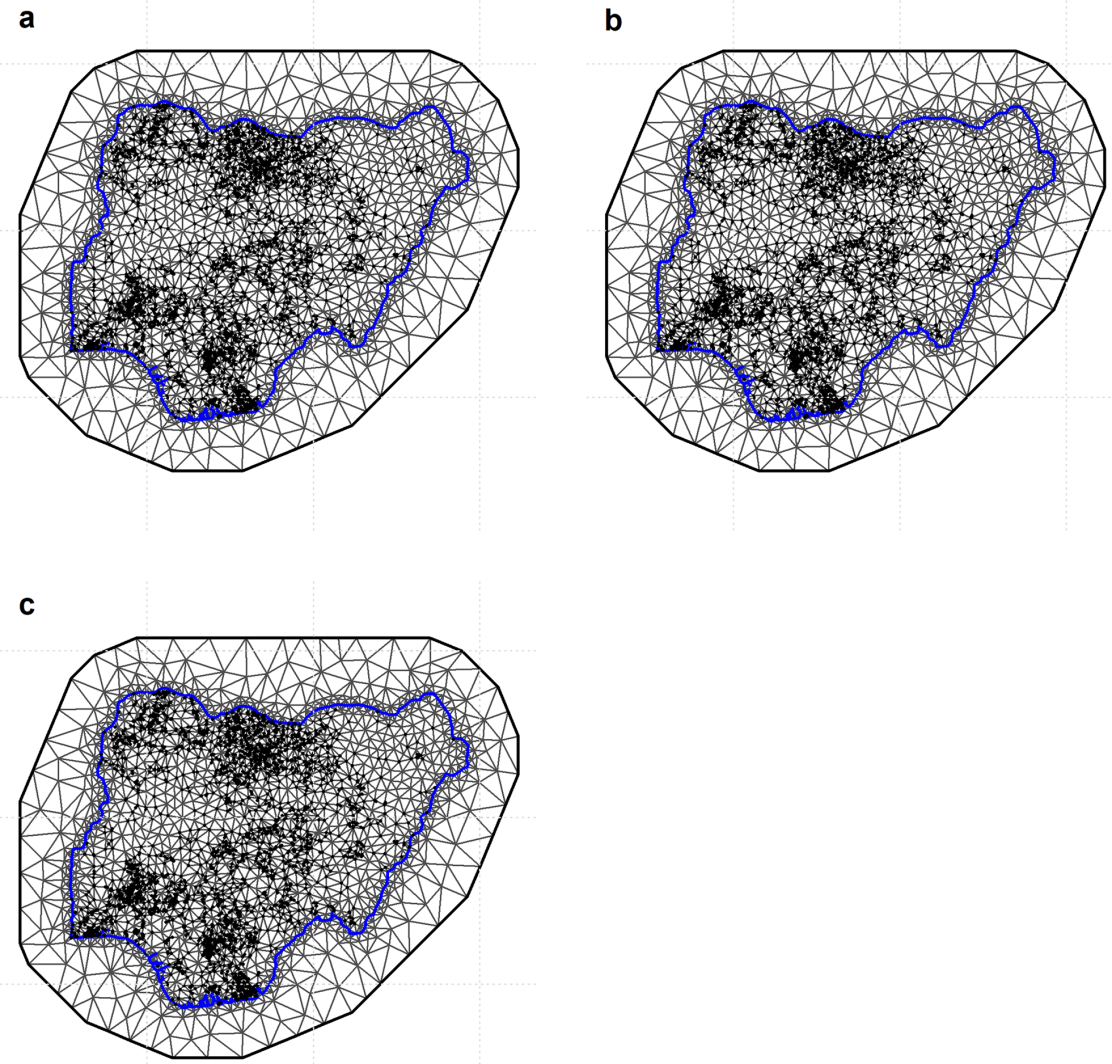
Triangulation of Nigeria based on** a** 2309 vertices (malaria and *Ascaris lumbricoides*). **b** 2311 vertices (malaria and hookworm). **c** 2305 vertices (malaria and *Trichuris trichiura*)

The triangulation maps in Fig. 2 show the mesh structures used for the spatial analysis of (a) malaria and *Ascaris lumbricoides*, (b) malaria and hookworm, and (c) malaria and *Trichuris trichiura * in Nigeria. These triangulations comprise 2309, 2311, and 2305 vertices respectively. The vertices are connected to form a network of triangular elements, establishing an artificial neighborhood structure that facilitates the modeling of spatial autocorrelation across the study area.

We analyzed the spatial effects of malaria and *Ascaris lumbricoides*, malaria and hookworm, and malaria and *Trichuris trichiura* in Nigeria, using the statistical software R 4.3.3 (R Foundation for Statistical Computing, Vienna, Austria). Insights were provided into the prevalence and incidence of these parasitic diseases across different regions of the country.

## Results

### Spatial correlation of malaria prevalence and STH prevalence and incidence

The posterior distributions of some of the parameters of the spatial fields in the model are summarized in Tables 2, 3, and 4. Malaria prevalence exhibits substantial spatial correlation with ranges extending between approximately 90 km and 350 km (1 decimal degree = 110.567 kms), indicating that its influence spans over large distances and shows broad spatial dependence. In contrast, *Ascaris lumbricoides*, hookworm, and *Trichuris trichiura* prevalence show more localized spatial effects, with ranges from about 43 km to 72 km, suggesting rapidly diminishing correlations over shorter distances. Meanwhile, *Ascaris lumbricoides*, hookworm, and *Trichuris trichiura* incidence presents moderate spatial extents, with ranges between approximately 92 km and 195 km, indicating a moderate level of spatial correlation suggesting a more contained spatial influence than malaria prevalence.

### Spatial relationships between malaria prevalence and STH prevalence and incidence

The spatial weight, $\lambda _1$, suggests an inverse relationship between malaria and the prevalence of *Ascaris lumbricoides*, hookworm, and *Trichuris trichiura*, indicating that STH prevalence tends to decrease as malaria prevalence increases. Meanwhile, the spatial weight, $\lambda _2$, shows a complex relationship: for *Ascaris lumbricoides* and hookworm, $\lambda _2$ suggests a direct but weak association with incidence, indicating a slight increase in *Ascaris lumbricoides* and hookworm incidence with rising malaria prevalence. However, for *Trichuris trichiura*, $\lambda _2$ indicates an inverse relationship, where an increase in malaria prevalence is associated with a decrease in *Trichuris trichiura* incidence.

### Spatial distribution of malaria prevalence and *Ascaris lumbricoides* prevalence and incidence

**Fig. 3:**
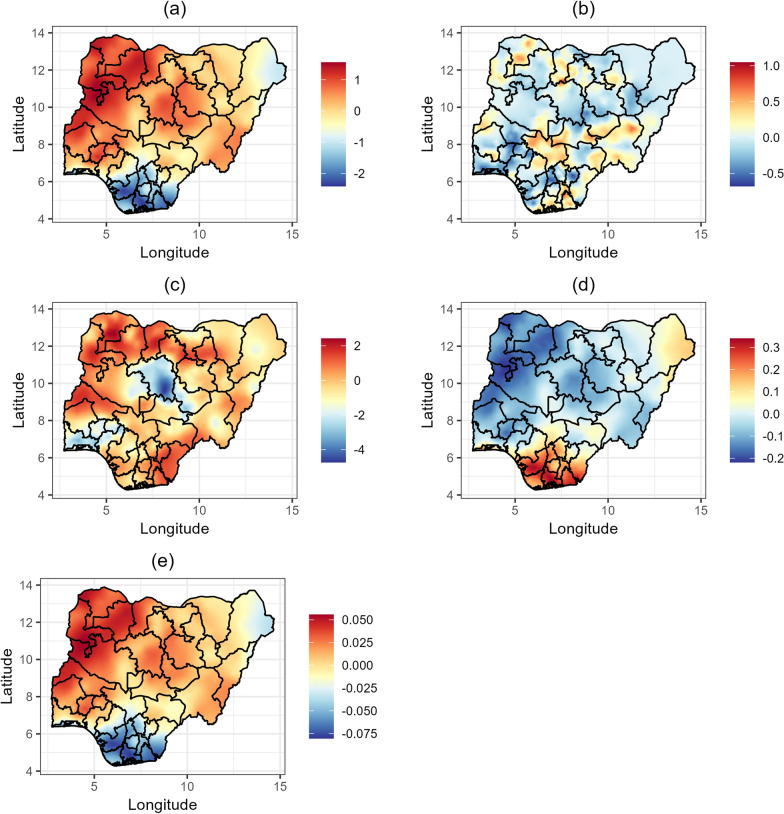
Estimates of the posterior means of spatial effects for **a** malaria prevalence. **b** Ascaris lumbricoides prevalence. **c** Ascaris lumbricoides incidence. **d** malaria prevalence and *Ascaris lumbricoides* prevalence. **e** malaria prevalence and *Ascaris lumbricoides* incidence

**Fig. 4:**
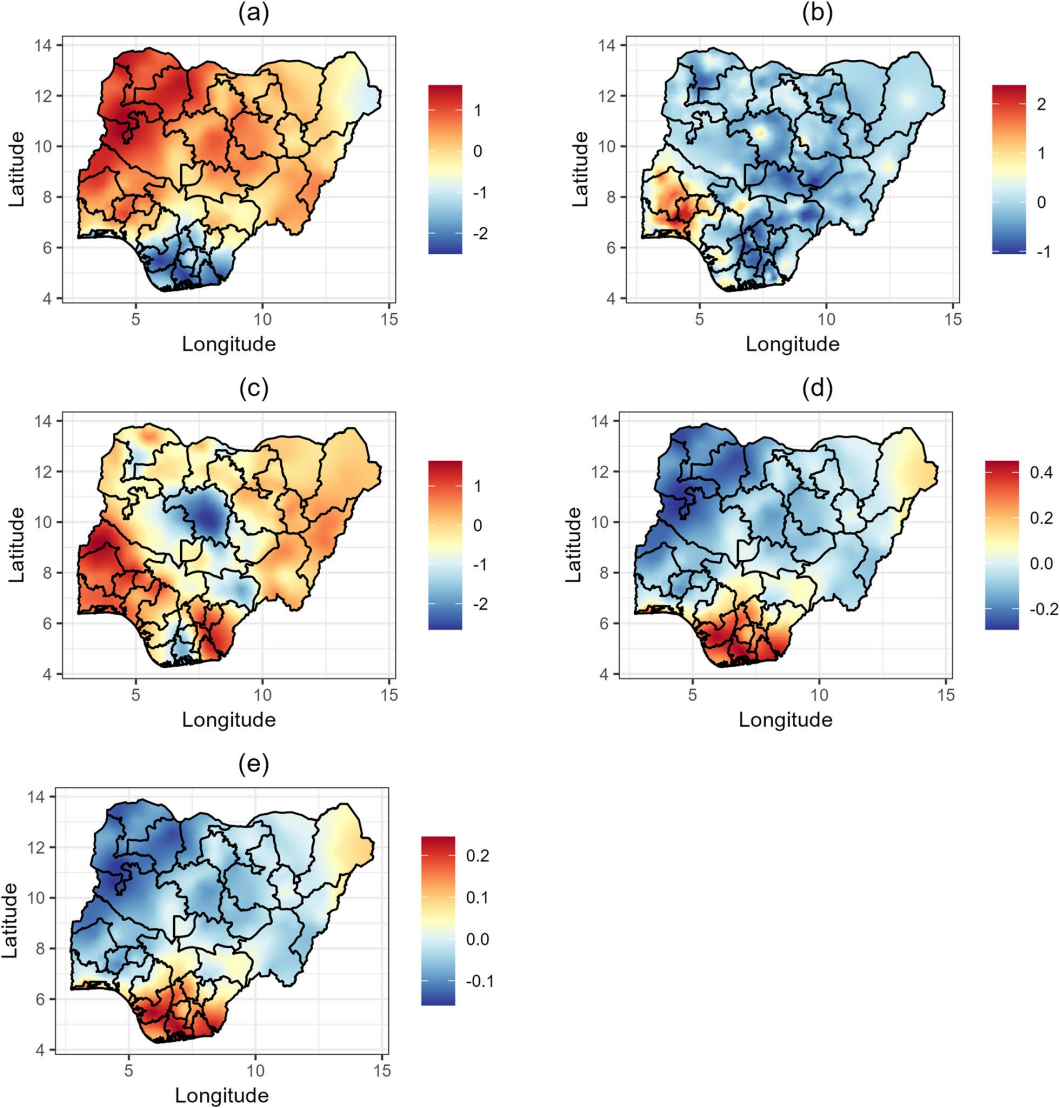
Estimates of the posterior means of spatial effects for malaria and hookworm. **a** malaria prevalence.** b** hookworm prevalence. **c** hookworm incidence. **d** malaria prevalence and hookworm prevalence.** e** malaria prevalence and hookworm incidence

**Fig. 5:**
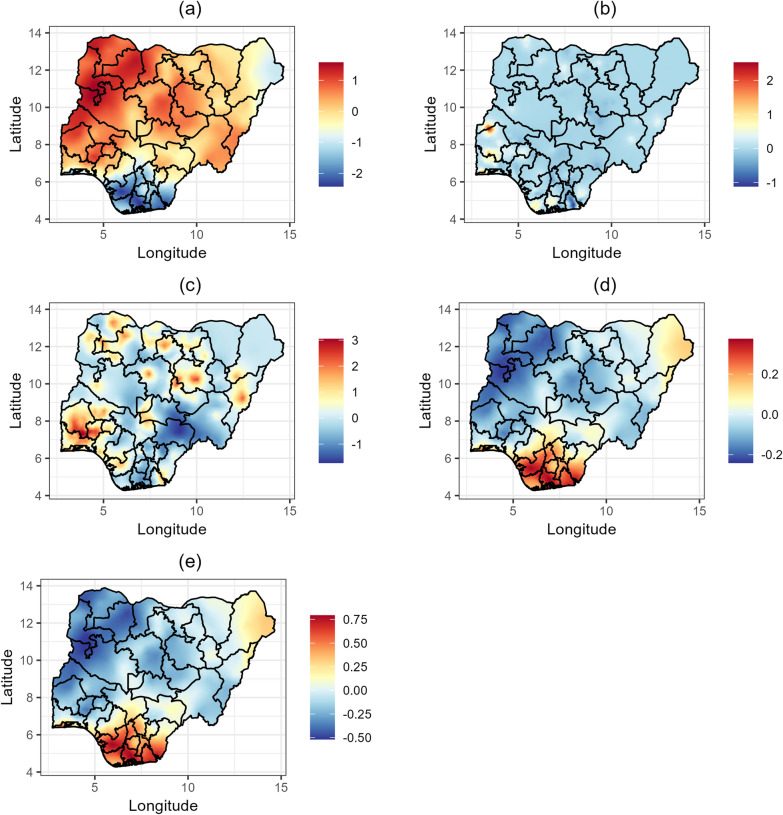
Estimates of the posterior means of spatial effects for malaria and *Trichuris trichiura*. **a** malaria prevalence. **b** *Trichuris trichiura* prevalence. **c** *Trichuris trichiura* incidence. **d** malaria prevalence and *Trichuris trichiura* prevalence.** e** malaria prevalence and *Trichuris trichiura* incidence

The posterior means of spatial effects for malaria prevalence, *Ascaris lumbricoides* prevalence, and *Ascaris lumbricoides* incidence, along with estimates of the shared spatial effects are presented in Fig. 3. The shared spatial effects represent areas where the spatial patterns of malaria and *Ascaris lumbricoides* overlap. Malaria prevalence (Fig. 3a) is highest in the northwestern states (Kebbi, Sokoto, Zamfara, and Katsina), parts of the northcentral states (Niger and Kwara), and some southwestern states (Oyo, Osun, and Ekiti). On the other hand, lower malaria prevalence is observed in the southeastern states (Enugu, Ebonyi, Anambra, Imo, and Abia) and in the southsouth (Cross River, Akwa Ibom, Rivers, and Bayelsa), as well as in parts of Borno. These findings are consistent with the results shown in Figs. 4a and 5a. Elevated *Ascaris lumbricoides* prevalence (Fig. 3b) is observed particularly in Rivers, Akwa Ibom, Taraba, Benue, Plateau, Nasarawa, Kogi, Oyo, Kwara, Niger, Kebbi, Sokoto, Katsina, Kano, Bauchi, and Adamawa. This distribution indicates significant prevalence across both southern and northern regions of the country. Figure 3c shows areas with frequent occurrences of *Ascaris lumbricoides* infections, with the highest incidence found in the northwest (Jigawa, Kaduna, Kano, Katsina, Kebbi, Sokoto, and Zamfara), the southsouth (Cross River, Akwa Ibom), and Ebonyi. Lower incidence rates are noted in the southwestern states (Lagos, Ogun, Osun, Ondo), Kaduna, and Plateau. Furthermore, the shared effect between malaria prevalence and *Ascaris lumbricoides* prevalence (Fig. 3d) shows higher co-morbidity in the southsouth (Edo, Delta, Bayelsa, Rivers, Akwa Ibom, and Cross River) and southeast (Imo, Abia, Ebonyi, Enugu, and Anambra), while prevalence is very low in the northwest. Conversely, the shared effect between malaria prevalence and *Ascaris lumbricoides* incidence (Fig. 3e) highlights the lowest occurrences in the southsouth and southeast, with the highest occurrences in the northwest.

### Spatial distribution of malaria prevalence and hookworm prevalence and incidence

The posterior means of spatial effects for malaria prevalence, hookworm prevalence, hookworm incidence, and the shared spatial effects are presented in detailed maps in Fig 4. Hookworm prevalence (Fig. 4b) is highest in the southwest (Oyo, Osun, Ekiti, Ogun, Lagos, Ondo). In comparison, hookworm incidence (Fig. 4c) varies significantly by region. Lower incidences are observed in Kaduna, Rivers, Benue, and some parts of Sokoto, Bayelsa, Delta, and Imo, while higher incidences are found in the southwest (Oyo, Osun, Ekiti, Ogun, Lagos, Ondo), the southsouth (Cross River, Akwa Ibom), and the southeast (Ebonyi, Abia, Imo). The shared effects (Fig. 4d and e) reveal higher co-morbidity between malaria and hookworm in the southsouth (Edo, Delta, Bayelsa, Rivers, Akwa Ibom, and Cross River), the southeast (Imo, Abia, Ebonyi, Enugu, and Anambra), and the southwest (Lagos), as well as some parts of Borno state, while co-morbidity is lowest in the northwest.

### Spatial distribution of malaria prevalence and *Trichuris trichiura* prevalence and incidence

The posterior means of spatial effects for malaria prevalence, *Trichuris trichiura* prevalence, *Trichuris trichiura* incidence, and the shared spatial effects are presented in detailed maps in Fig. 5. Figure 5b reveals that among all STHs, *Trichuris trichiura* is the least prevalent in Nigeria, with small but noticeable prevalence in Oyo, Kwara, Ogun, Lagos, Bayelsa, and Rivers states. *Trichuris trichiura* incidence (Fig. 5c) shows significant regional variations. The lowest incidence rates are observed in Adamawa, Taraba, Benue, Ebonyi, Enugu, Imo, Rivers, and Gombe states. Conversely, higher incidences are found in Oyo, Ogun, Lagos, and some areas in Sokoto, Kebbi, Zamfara, Katsina, Kano, Jigawa, Bauchi, Borno, and Adamawa states. The shared effects (Fig. 5d and e) also show higher co-morbidity between malaria and *Trichuris trichiura* in the southsouth (Edo, Delta, Bayelsa, Rivers, Akwa Ibom, and Cross River), the southeast (Imo, Abia, Ebonyi, Enugu, and Anambra), the southwest (Lagos), and some parts of Borno state, while co-morbidity is lowest in the northwest.

### Climate impact on malaria and STH

**Fig. 6:**
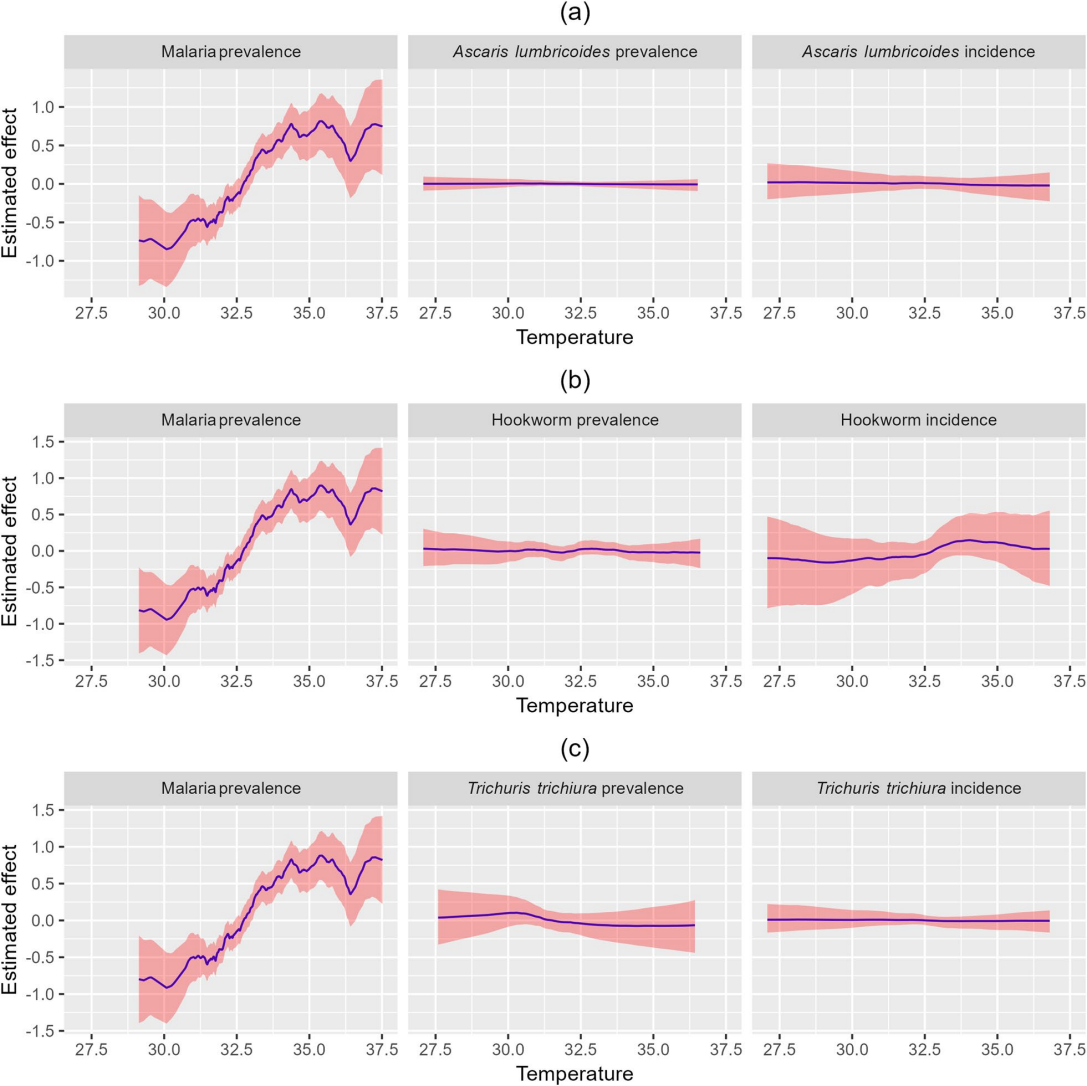
Effects of temperature on the spatial distribution of malaria prevalence and STH prevalence and incidence

**Fig. 7:**
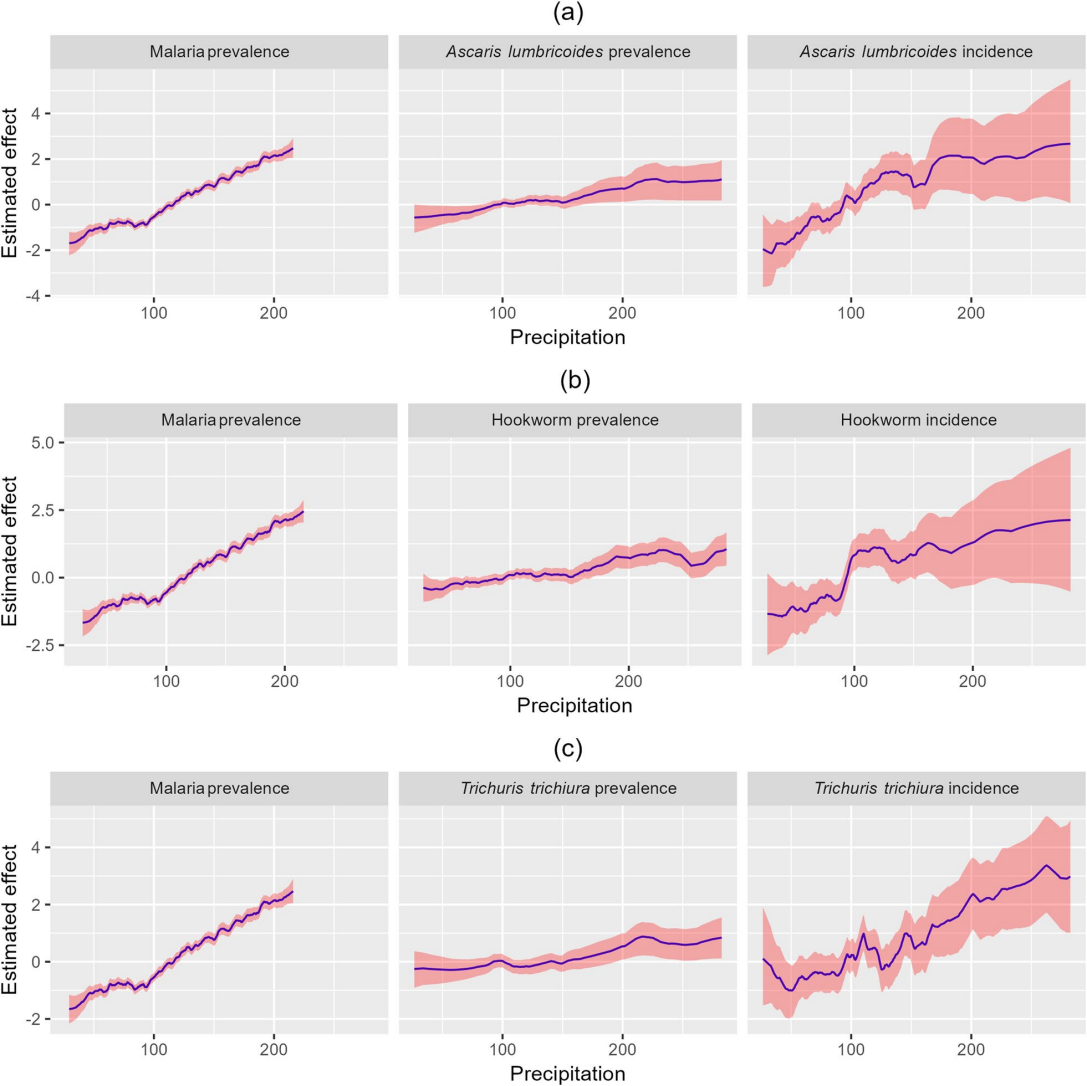
Effects of precipitation on the spatial distribution of malaria prevalence and STH prevalence and incidence

The impact of temperature and precipitation on the distribution of malaria and STH is illustrated in Figs. 6 and 7. There is a strong positive relationship between temperature and malaria prevalence, with the estimated effect increasing sharply, particularly after temperature exceeds 30$^\circ$C. This suggests that higher temperature is closely linked to increased malaria prevalence, and the effect becomes more pronounced as temperature rises (Fig. 6a, b, c). In contrast, *Ascaris lumbricoides* incidence and prevalence show minimal to no relationship with temperature (Fig. 6a). Similarly, hookworm prevalence is minimally affected by temperature (Fig. 6b), and *Trichuris trichiura* infections show little to no impact from temperature, with estimated effects consistently close to zero (Fig. 6c), indicating that temperature is not a significant factor in STH transmission. On the other hand, higher precipitation is associated with increased malaria prevalence as well as higher STH prevalence and incidence (Fig. 7).

**Table 2:** Summary of the posterior distributions for parameters in the model describing the interactions between malaria prevalence and *Ascaris lumbricoides* prevalence and incidence

Parameter	Mean	SD	2.5%	97.5%
${\alpha }_{1}$	− 0.700	0.324	− 1.334	− 0.026
${\alpha }_{2}$	− 2.018	0.117	− 2.246	− 1.778
${\alpha }_{3}$	0.738	0.316	0.123	1.371
Stdev 1	0.819	0.112	0.620	1.059
Stdev 2	0.649	0.057	0.548	0.772
Stdev 3	1.136	0.167	0.832	1.490
Range 1	3.163	0.585	2.166	4.461
Range 2	0.963	0.149	0.724	1.309
Range 3	1.970	0.574	1.099	3.340
${\lambda }_{1}$	− 0.144	0.110	− 0.363	0.069
${\lambda }_{2}$	0.097	0.246	− 0.379	0.590
${\lambda }_{3}$	1.350	0.244	0.876	1.836

The table includes the estimated mean, SD, and credible intervals for each parameter associated with each outcome

**Table 3:** Summary of the posterior distributions of some parameters from the model for malaria prevalence and hookworm prevalence and incidence

Parameter	Mean	SD	2.5%	97.5%
${\alpha }_{1}$	− 0.733	0.325	1.380	− 0.074
${\alpha }_{2}$	− 1.900	0.063	− 2.022	− 1.774
${\alpha }_{3}$	2.004	0.327	1.376	2.675
Stdev 1	0.708	0.099	0.508	0.890
Stdev 2	0.385	0.033	0.322	0.451
Stdev 3	1.765	0.185	1..440	2.169
Range 1	2.702	0.485	1.745	3.615
Range 2	0.844	0.169	0.610	1.263
Range 3	1.661	0.271	1.229	2.291
${\lambda }_{1}$	− 0.192	0.068	− 0.325	− 0.058
${\lambda }_{2}$	0.005	0.163	− 0.331	0.314
${\lambda }_{3}$	0.305	0.282	− 0.264	0.846

The table includes the estimated mean, SD, and credible intervals for each parameter associated with each outcome

**Table 4:** Summary of the posterior distributions of some parameters from the model for malaria prevalence and *Trichuris trichiura* prevalence and incidence

Parameter	Mean	SD	2.5%	97.5%
${\alpha }_{1}$	− 0.727	0.300	− 1.318	− 0.136
${\alpha }_{2}$	− 2.666	0.115	− 2.949	− 2.473
${\alpha }_{3}$	− 2.420	0.215	− 2.844	− 1.997
Stdev 1	0.815	0.114	0.616	1.064
Stdev 2	0.605	0.066	0.485	0.743
Stdev 3	1.253	0.146	0.996	1.568
Range 1	3.316	0.592	2.416	4.468
Range 2	0.482	0.123	0.284	0.766
Range 3	0.829	0.197	0.506	1.278
${\lambda }_{1}$	− 0.133	0.128	− 0.388	0.117
${\lambda }_{2}$	− 0.308	0.197	− 0.691	0.083
${\lambda }_{3}$	0.358	0.328	− 0.278	1.013

The table includes the estimated mean, SD, and credible intervals for each parameter associated with each outcome

## Discussion

This research comprehensively investigates the geographical patterns and climatic determinants of co-morbidity between malaria and soil-transmitted helminths (STH) in Nigeria. Given that both diseases primarily affect children, often compounding health issues and hindering development, the study is important as it aids in identifying locations where each disease is most likely to occur, their severity at different locations, as measured by prevalence, and the co-morbidity between the two.

Our findings reveal a consistent pattern of elevated malaria prevalence in the northwest and north-central regions, contrasting sharply with lower rates observed in the southern states and parts of Borno. These geographic variations likely reflect underlying environmental conditions, such as temperature and humidity, as well as differences in the effectiveness and reach of health interventions. According to the results in Tables 1, 2, and 3, malaria prevalence exhibits substantial spatial correlation, with ranges extending between approximately 90 km to 350 km (1 decimal degree = 110.567 kms), indicating its influence spans over large distances and shows broad spatial dependence. Previous studies have reported similar disparities in malaria prevalence across Nigeria, emphasizing the persistently high burden in the northern regions [42]. This is further supported by [21], who documented significant malaria prevalence in these areas, reinforcing the need for targeted malaria control strategies. Focused interventions, such as the distribution of insecticide-treated nets, indoor residual spraying, and improved access to effective antimalarial treatments, are essential to address the high burden of malaria in these regions.

Among all STHs, *Ascaris lumbricoides* is particularly prevalent in both southern and northern Nigeria, making it the most widespread STH in the country. This widespread nature can be attributed to several factors, including poor socio-economic conditions, inadequate sanitation, and cultural practices such as open defecation and geophagia [43]. However, hookworm infections exhibit a distinct geographical distribution, with the highest prevalence found in the southwest. Although *Trichuris trichiura* is also most common in the southwest, it is less prevalent overall than *Ascaris lumbricoides* and hookworm, ranking as the least prevalent STH in Nigeria. Nonetheless, *Trichuris trichiura* poses a significant public health concern; even at lower prevalence rates, it can lead to considerable morbidity. Furthermore, STH prevalence shows more localized spatial effects, with ranges from about 43 km to 72 km, suggesting rapidly diminishing correlations over shorter distances. According to [44], the southwestern region of Nigeria may be the most endemic for STHs due to its forested environment, high rainfall, and specific climatic conditions that facilitate the survival and transmission of these parasites. Improving water, sanitation, and hygiene (WASH) infrastructure with regular deworming campaigns can help reduce STH infections.

Interestingly, the spatial weight, $\lambda _1$, reflects an inverse relationship between malaria and STH prevalence, indicating that STH prevalence tends to decrease as malaria prevalence increases. A previous study indicated that while STH contributes to an increase in the incidence of clinical malaria, it has minimal impact on its severity [45]. However, it is important to interpret this relationship with caution, especially given the methodological limitations of ESPEN surveys. These surveys are not as robust in their sampling approaches as those used for DHS surveys, and comprehensive state-level data were unavailable for many states in Nigeria during the period from 1978 to 2014. Furthermore, while malaria control programs are plausible to indirectly reduce STH transmission through improvements in sanitation and hygiene, this explanation may not fully account for the observed trend. Report indicate that while 70% of Nigerians have access to basic water services, significant challenges remain, including poor water quality, widespread open defecation, and limited access to handwashing facilities [46]. These gaps in sanitation and hygiene hinder the potential role of hygiene interventions in driving the decline in STH prevalence.

However, significant overlaps in co-morbidity between malaria and STH were identified, particularly in the south-south and southeast regions, where both diseases exhibit high prevalence. In contrast, the northwest shows lower co-morbidity despite higher malaria prevalence. This co-occurrence underscores the compounded health burden in the southern region of Nigeria, necessitating integrated disease management approaches that encompass community education on hygiene practices and regular health screenings for both diseases.

Climate change negatively impacts human health, exacerbating health risks [47]. Research has shown that rising ambient temperatures, a consequence of climate change, significantly contribute to increased morbidity rates in Nigeria, identifying cholera, meningitis, malaria, and pneumonia as primary health risks intensified by these changes [47, 48]. Our study corroborates these findings, demonstrating that temperature plays a critical role in malaria transmission, with a marked increase in prevalence observed as temperature exceeds 30 $^\circ$C, suggesting that higher temperatures can accelerate the development of malaria parasites within mosquito vectors, thereby enhancing their transmission potential.

Temperature is one variable that has traditionally been viewed as a significant factor affecting STH dynamics [49, 50]. The impact of climate change on malaria and neglected tropical diseases varies depending on the specific disease and geographic area [51]. Our results indicate that temperature has a minimal direct effect on STH prevalence and incidence in Nigeria. This suggests that other factors, such as sanitation practices, soil characteristics, and environmental conditions, may play a more substantial role in STH transmission. As noted by [52], the persistence of helminth infections is closely tied to favorable soil conditions and regular contamination of the environment through human waste such including open defecation, which is still commonly practiced in most parts of Nigeria [53]. Thus, public health initiatives must focus on improving these determinants to effectively combat STH.

Contrarily, precipitation emerges as a crucial factor positively correlating with both malaria and STH prevalence and incidence. Increased rainfall creates optimal breeding conditions for malaria vectors and supports the environmental persistence of STH eggs, thereby enhancing transmission rates [54]. These findings underscore the critical need for public health interventions that are sensitive to climatic influences, particularly in regions where the interplay between environmental factors and disease transmission dynamics is pronounced.

Despite the valuable insights gained, our study has some limitations. The reliance on secondary data from diverse sources may introduce biases that affect the accuracy of co-morbidity prevalence estimates. While we identified climatic factors, we did not thoroughly examine other determinants of disease transmission, such as socioeconomic status, sanitation practices, and health infrastructure. Moving forward, future research should explore historical and projected climate change scenarios to assess their effects on the distribution and prevalence of malaria and STH [51]. Investigating climate change mitigation and adaptation strategies, such as improving sanitation infrastructure and implementing effective vector control measures, could provide critical insights into reducing the disease burden in affected populations. Also, future studies should incorporate a broader range of covariates, employ spatiotemporal analysis, and examine how environmental and socioeconomic changes influence disease dynamics over time.

## Conclusions

This research underscores the significant spatial variations in the prevalence of malaria and STH across Nigeria and their co-morbidity, highlighting the need for tailored public health interventions. By identifying the unique disease burdens and co-morbidity patterns in different regions, targeted strategies can be developed to enhance health outcomes for affected populations. Our study also emphasizes the significant influence of climatic factors on the transmission dynamics of both diseases. Temperature accelerates malaria transmission by enhancing parasite development in mosquitoes, while its effect on STH is minimal, with sanitation and soil conditions playing a more prominent role. Precipitation, on the other hand, is positively correlated with both malaria and STH, promoting mosquito breeding and the persistence of STH eggs. These findings underscore the need for climate-sensitive approaches to disease control. The insights gained from this study should inform future research efforts and guide policy-making. Specifically, integrated approaches to combat malaria and STH are essential, particularly in light of the ongoing challenges posed by climate change. A collaborative effort that combines health initiatives with environmental considerations will be vital for fostering sustainable health improvements.

## Data Availability

The datasets analyzed during the current study include publicly available data from the Demographic and Health Surveys (DHS) and the Expanded Special Project for Elimination of Neglected Tropical Diseases (ESPEN) portal. These datasets can be accessed directly online.
